# Metabolic PET/CT analysis of aggressive Non-Hodgkin lymphoma prior to Axicabtagene Ciloleucel CAR-T infusion: predictors of progressive disease, survival, and toxicity

**DOI:** 10.1038/s41408-023-00895-7

**Published:** 2023-08-18

**Authors:** William G. Breen, Jason R. Young, Matthew A. Hathcock, Roman O. Kowalchuk, Matthew P. Thorpe, Radhika Bansal, Arushi Khurana, N. Nora Bennani, Jonas Paludo, Jose Villasboas Bisneto, Yucai Wang, Stephen M. Ansell, Jennifer L. Peterson, Patrick B. Johnston, Scott C. Lester, Yi Lin

**Affiliations:** 1 Department of Radiation Oncology, Mayo Clinic, Rochester, MN USA; 2 Department of Radiology, Mayo Clinic, Rochester, MN USA; 3 Department of Biomedical Statistics and Informatics, Mayo Clinic, Rochester, MN USA; 4 Division of Hematology, Department of Medicine, Mayo Clinic, Rochester, MN USA; 5 Department of Radiation Oncology, Mayo Clinic, Jacksonville, FL USA

**Keywords:** Radiotherapy, Cancer metabolism, Cancer imaging

## Abstract

PET/CT is used to evaluate relapsed/refractory non-Hodgkin lymphoma (NHL) prior to chimeric antigen receptor T-cell (CAR-T) infusion at two time points: pre-leukapheresis (pre-leuk) and pre-lymphodepletion chemotherapy (pre-LD). We hypothesized that changes in PET/CT between these time points predict outcomes after CAR-T. Metabolic tumor volume (MTV), total lesion glycolysis (TLG), and other metrics were calculated from pre-leuk and pre-LD PET/CT scans in patients with NHL who received axicabtagene ciloleucel, and assessed for association with outcomes. Sixty-nine patients were analyzed. While single time point PET/CT characteristics were not associated with risk of PD or death, increases from pre-leuk to pre-LD in parenchymal MTV, nodal MTV, TLG of the largest lesion, and total number of lesions were associated with increased risk of death (*p* < 0.05 for all). LASSO analysis identified increasing extranodal MTV and increasing TLG of the largest lesion as strong predictors of death (AUC 0.74). Greater pre-LD total MTV was associated with higher risk of grade 3+ immune effector cell-associated neurotoxicity syndrome (ICANS) (*p* = 0.042). Increasing metabolic disease burden during CAR-T manufacturing is associated with increased risk of progression and death. A two variable risk score stratifies prognosis prior to CAR-T infusion and may inform risk-adapted strategies.

## Introduction

Chimeric antigen receptor T-cell (CAR-T) therapy has demonstrated promising outcomes for patients with aggressive relapsed or refractory B-cell non-Hodgkin lymphoma (NHL) and provides an opportunity for long-term remission [1–5]. However, given the considerable potential toxicities, costs, and rates of disease progression following CAR-T therapy, advancements in risk stratification are needed to further tailor patient selection and treatment modification.

In clinical practice, F-18 fluorodeoxyglucose positron emission tomography computed tomography (PET/CT) is used to evaluate disease extent prior to CAR-T infusion at two time points: pre-leukapheresis (pre-leuk) approximately 6 weeks prior to CAR-T infusion, and pre-lymphodepletion chemotherapy (pre-LD) approximately 1 week prior to CAR-T infusion. PET/CT characteristics and changes in these characteristics may assist in response-adapted treatment and monitoring strategies including the identification of patients who would benefit from intensification of treatment or other modifications to the treatment plan [6, 7].

Despite advancements in PET/CT imaging and analysis, current standards divide disease status into Deauville scores ranging from 1 to 5. Within these broad categories there is considerable variability in biology and prognosis. Recent studies have demonstrated the favorable prognostic value of volumetric metabolic PET/CT characteristics in lymphoma, including metabolic tumor volume (MTV) and total lesion glycolysis (TLG) [8–14]. These metabolic data represent opportunities for PET/CT to provide more accurate prognosis and guide therapy.

In patients receiving CAR-T therapy, it is unknown whether individual time point pre-leuk and pre-LD PET/CT characteristics are predictive of outcomes, or whether changes between time points are more prognostic. We aimed to develop a simple tool using pre-leuk and pre-LD PET/CT characteristics, or changes in these characteristics between time points, to stratify patients by predicted OS after CAR-T in order to guide further management and research.

## Materials/subjects and methods

### Patients

A prospectively maintained institutional database of patients receiving axicabtagene ciloleucel (axi-cel) CAR-T therapy was utilized to identify patients. All patients 18 years and older with relapsed/refractory, aggressive NHL treated with CAR-T were included in this analysis. Bridging therapy was given per the treating physician’s discretion to control or debulk disease, typically due to concern for symptomatic or potentially life-threatening progression during manufacturing in the absence of therapy. Infusion and post-infusion management were based on institutional protocols.

### PET analysis and patient assessments

**Fig. 1:**
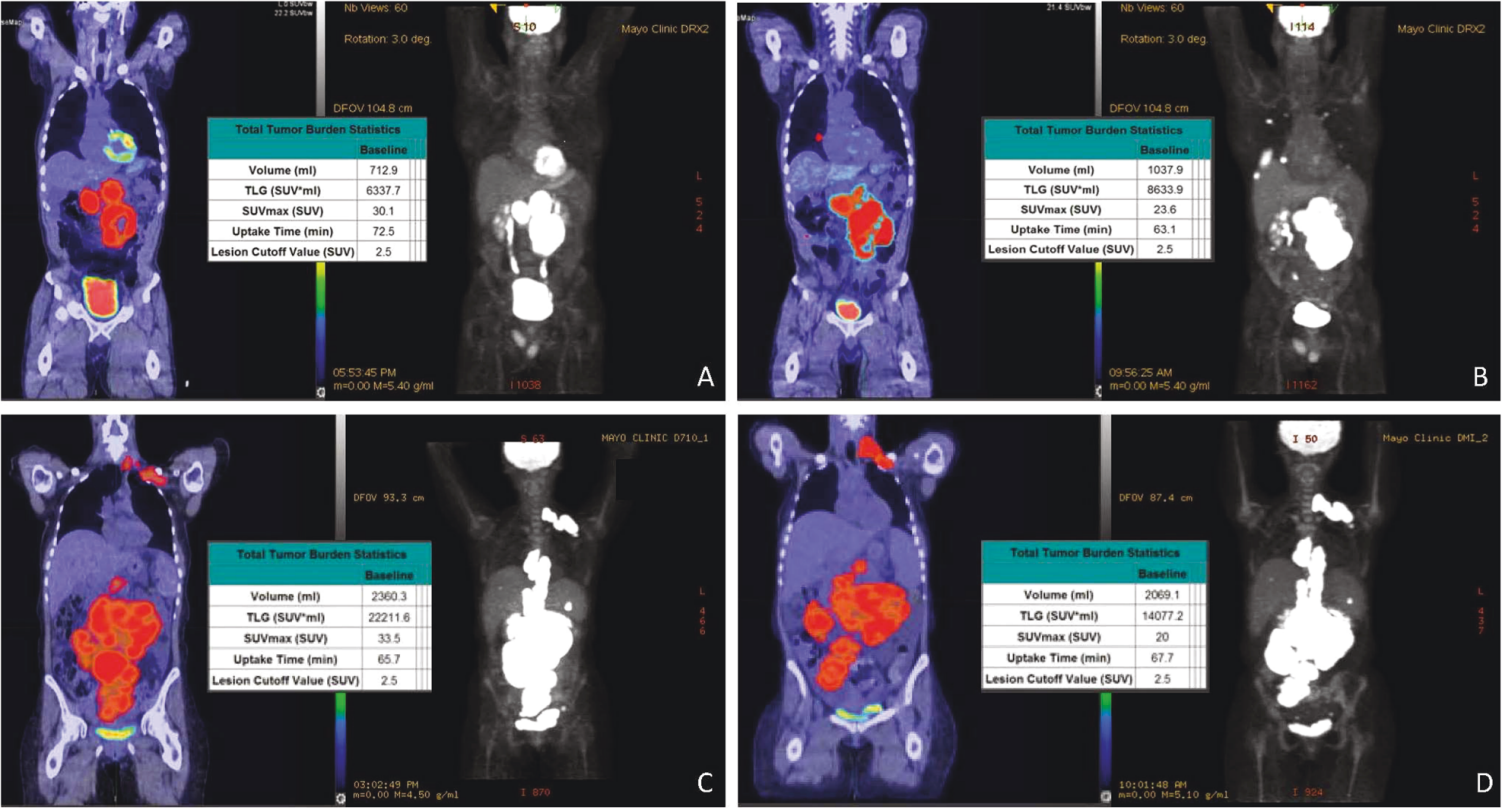
Representative Cases Demonstrating Metabolic FDG PET/CT Analysis. Pre-leukapheresis (pre-leuk) and pre- lymphodepletion chemotherapy (pre-LD) coronal fused PET/CT (left) and maximum projection image (right) PET/CT imaging and total tumor volumetric analysis for two patients. The first patient **A**, **B** had both increasing extranodal metabolic tumor volume (MTV) and increasing total lesion glycolysis (TLG) of the largest lesion (risk score 2) from pre-leuk **A** to pre-LD **B**, and experienced PD and death after CAR-T infusion. The second patient **C**, **D** had neither risk factor (risk score 0), is alive and without progression at last follow-up, despite having higher MTV and TLG at each individual time point compared to the first patient.

At both pre-leuk and pre-LD time points, FDG PET/CT was used to assess extent of disease. In real time, Lugano criteria was used to classify disease status [15]. Retrospectively, lesions from pre-leuk and pre-LD PET/CT scans were segmented with a fixed absolute standard uptake value maximum (SUVMax) threshold of 2.5 using a semi-automated workflow (LesionID, MIM Software Inc., Cleveland, Ohio, USA) with manual modification to exclude physiologic uptake as needed [8]. MTV, TLG, SUVMax, mean SUV, number, and anatomic location of all lymphomatous lesions were assessed for each PET/CT, and changes from pre-leuk to pre-LD were also calculated (examples shown in Fig. 1). Lesions were categorized as either nodal, spleen, bone, parenchyma (e.g., liver, lung), or soft tissue (e.g., subcutaneous, muscle), and MTV was calculated for each category. The mesenteric disease was defined as nodal. Discrete, avid bone lesions were contoured and included, but diffuse uptake indistinguishable from marrow was not included. Non-nodal categories were aggregated as “extranodal.”

Following CAR-T infusion, patients continued routine clinical follow up including PET/CT surveillance at one-month, then three-month intervals. PD was defined using Lugano criteria, with biopsy confirmation when clinically indicated. Initiation of further salvage treatments was at physician discretion.

### Statistical analyses

Baseline patient characteristics and PET/CT parameters were summarized as counts and percentages, median and interquartile range, or mean and standard deviation. When appropriate, continuous PET values were dichotomized to create scientifically appropriate groups (e.g., SUVMax <10 vs. ≥10, bone MTV 0 cc vs. >0 cc). Otherwise, maximally selected rank statistics were used to establish an optimal cutoff point to create categorical variables.

Overall survival (OS) and event-free survival (EFS) were calculated using the Kaplan-Meier method from the time of CAR-T infusion. For EFS, any progression, initiation of salvage therapy, or death were considered an event. Univariate Cox modeling was used to associate relative and directional change in metabolic and volumetric PET/CT characteristics with PD and death, after adjusting for bridging therapy. LASSO (least absolute shrinkage and selection operator) method was used for multivariable model selection, adjusting for bridging as a fixed effect. This regression method uses both variable selection and regularization to optimize the accuracy and interpretability of results. Candidate variables assessed at each timepoint (and assessed for changes between time points) are listed in Supplemental Table 1. Pre-LD PET/CT characteristics were also assessed for association with the presence and duration of grade 3+ immune effector cell-associated neurotoxicity syndrome (ICANS), duration of cytokine release syndrome (CRS), tocilizumab use, and corticosteroid use. Analyses were conducted using R (Version 3.6.3, R Foundation for Statistical Computing, Vienna, Austria). For all analyses, a *p*-value < 0.05 was considered statistically significant.

## Results

Sixty-nine patients with NHL were treated with axi-cel CAR-T therapy between January 2018 and July 2020 (Table 1). Pre-leuk and pre-LD PET/CT scans were performed a median of 46 days and 7 days prior to CAR-T infusion, respectively.

**Table 1:** Patient and treatment characteristics.

Patients		*N* = 69 (100%)
Age at CAR-T infusion	Median (years)	61 (range 26–76)
Sex	Female	26 (38%)
Disease Subtype	Diffuse Large B-Cell Lymphoma	39 (57%)
	Transformed Follicular Lymphoma	16 (23%)
	High-grade Lymphoma	13 (19%)
	Primary Mediastinal B-Cell Lymphoma	1 (1%)
Previous Lines of Therapy	2	14 (20%)
	3	29 (42%)
	≥4	26 (38%)
B Symptoms at CAR-T Evaluation	Present	7 (10%)
	Absent	61 (88%)
	Unknown	1 (1%)
LDH Level at CAR-T Evaluation	Elevated	19 (28%)
	Normal	48 (70%)
	Not Evaluated	2 (3%)
CRP at CAR-T Infusion	>100 mg/L	6 (9%)
	10-100 mg/L	42 (61%)
	≤10 mg/L	21 (30%)
Ferritin at CAR-T Infusion	Elevated	25 (36%)
	Normal	44 (64%)
Received Bridging Therapy	Yes	44 (64%)
	No	25 (36%)

With a median follow-up of 13.3 months (interquartile range 4.7–18.0 months), the OS at 6, 12, and 18 months was 75%, 65%, and 42%, respectively. EFS at 6, 12, and 18 months was 43%, 39%, and 37%, respectively. Cumulative incidence of progression at 6, 12, and 18 months was 55%, 59%, and 62%, respectively. At the last follow-up, 46 patients (67%) had experienced PD and 30 patients (43%) had died.

When assessing individual pre-leuk and pre-LD PET/CT characteristics (i.e., single static time point, no change between time points), no variables including MTV, TLG, or SUVMax were associated with increased risk of PD. However, increases from pre-leuk to pre-LD in total MTV (HR: 1.16, 95% CI: 1.00–1.34, *p* = 0.048), total TLG (HR: 1.18, 95% CI: 1.02–1.38, *p* = 0.028), parenchymal MTV (HR: 3.72, 95% CI: 1.49–9.29, *p* = 0.005), and nodal MTV (HR: 1.23, 95% CI: 1.05–1.43, *p* = 0.010) were associated with increased risk of PD.

Similarly, no static time point pre-leuk or pre-LD PET/CT characteristics were associated with the risk of death. However, increases from pre-leuk to pre-LD in parenchymal MTV (HR 2.89, 95% CI: 1.17–7.09, *p* = 0.020), nodal MTV (HR: 1.20, 95%CI: 1.02–1.40, *p* = 0.030), TLG of the largest lesion (HR: 2.42, 95% CI: 1.021–5.73, *p* = 0.045), and total number of lesions (HR: 1.57, 95% CI: 1.13–2.18, *p* = 0.008) were associated with increased risk of death.

Forty-four (64%) of patients received bridging therapy. Patients receiving bridging had worse OS (*p* = 0.004), but significantly decreased rates of CRS and ICS post infusion (CRS: 62% v 30%, ICANS: 41% v 15%, *P*-value < 0.02). The duration of CRS and ICANS were not significantly different for patients who received bridging compared to those who did not receive bridging.

**Fig. 2:**
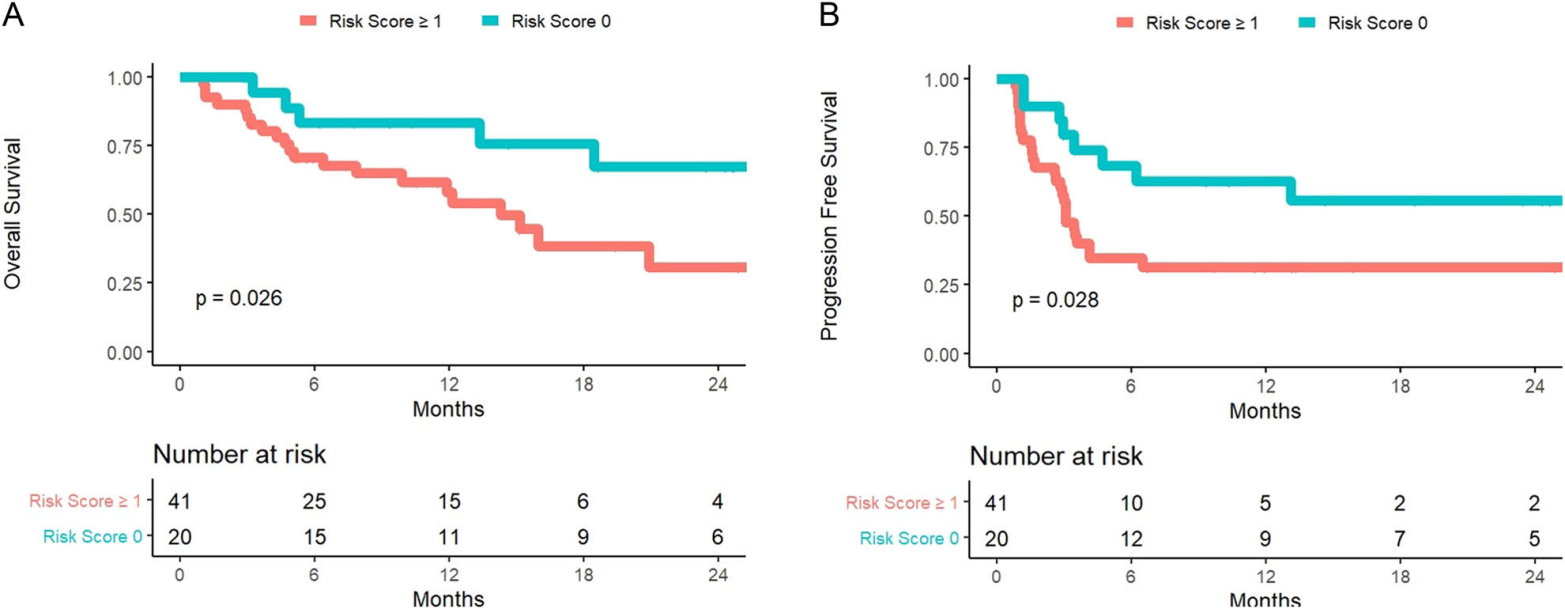
Overall Survival and Progression-Free Survival after CAR-T Infusion by Risk Score. Overall Survival **A** and Progression-Free Survival **B** according to a novel risk score based on the presence of either increasing extranodal metabolic tumor volume (MTV) or increasing total lesion glycolysis (TLG) of the largest lesion (risk score 1) versus neither risk factor (risk score 0). TLG incorporates both volume and degree of PET avidity from a lesion, and may act as a metabolic measure of tumor bulk.

LASSO analysis identified increasing extranodal MTV (≥25% increase) and increasing TLG of the largest lesion (≥10% increase) as strong predictors of death (AUC 0.74, Table 2), stratified for bridging therapy as a fixed effect. Kaplan-Meier plots were generated for overall and progression-free survival using these risk factors (Fig. 2A, B). In total, 36% of patients had neither increasing extranodal MTV or increasing TLG of the largest lesion, 24% had increasing TLG of the largest lesion but not increasing extranodal MTV, 22% had increasing extranodal MTV but not increasing TLG of the largest lesion, and 19% had both increasing extranodal MTV and increasing TLG of the largest lesion.

**Table 2:** Estimated risk of death at 12 months.

Increasing Extranodal MTV	Increasing TLG of the largest lesion	No Bridging	Bridging
No	No	5%	26%
No	Yes	8%	28%
Yes	No	14%	57%
Yes	Yes	21%	75%

Sixty patients (87%) developed CRS following CAR-T infusion for a median duration of 5 days. The presence of pre-LD parenchymal disease was associated with CRS duration greater than 5 days (HR: 3.82, 95% confidence interval (CI): 1.25–12.50, *p* = 0.021). Other PET/CT characteristics were not associated with longer duration CRS. Thirty-seven patients (54%) developed ICANS for a median duration of 4.5 days, including 12 (32%) with grade 3 + ICANS. Greater pre-LD total MTV was associated with higher risk of grade 3 + ICANS (HR 1.66, 95% CI: 1.07–2.87, *p* = 0.042). Unlike pre-LD PET/CT characteristics, there were no pre-leuk PET/CT characteristics associated with CRS or ICANS. Nineteen (28%) patients required tocilizumab. Greater pre-LD total MTV (HR: 1.67, 95% CI: 1.15–2.63, *p* = 0.014), TLG (HR: 1.54, 95% CI: 1.10–2.28, *p* = 0.18), and volume of the largest lesion (HR: 1.61, 95% CI: 1.12–2.48, *p* = 0.017) were associated with increased use of tocilizumab. Greater pre-LD total MTV (HR: 1.44, 95% CI: 1.06–2.11, *p* = 0.038) and TLG of the largest lesion (HR: 1.35, 95% CI: 1.02–1.86, *p* = 0.043) were associated with increased use of corticosteroid.

## Discussion

This study demonstrates that patients with increasing metabolic characteristics from pre-leuk to pre-LD PET/CT have increased risk of progression and death. We present a two variable risk score, adjusting for bridging therapy as a fixed effect, to account for prognosis prior to CAR-T infusion. As CAR-T therapy utilization and indications increase, methods of prognostic differentiation using non-invasive methods such as PET/CT are needed to identify which patients are at the highest risk of PD, death, and post-infusion toxicity, thereby indicating which patients may benefit from treatment intensification, increased monitoring, or prophylaxis of expected toxicities [2, 5, 16, 17].

The two variables predictive of death and incorporated into the model presented in this study are increasing extranodal MTV and increasing TLG of the largest lesion from pre-leuk to pre-LD PET/CT. The prognostic value of these variables appeared to hold whether patients did (64%) or did not (36%) receive bridging therapy between pre-leuk and pre-LD timepoints. Extranodal disease has long been established as a negative prognostic factor [18]. TLG incorporates both the size and metabolic activity of a lesion by multiplying MTV by mean SUV. Accordingly, the TLG of the largest lesions may be a metabolic analog of tumor bulk. Tumor bulk is a negative prognostic factor and was historically defined as greater than 1/3 the thoracic diameter of a chest x-ray [19]. As three-dimensional imaging was incorporated into clinical practice, the definitions of bulk evolved to a single axis lesion measurement of >6–10 cm on CT scans [20]. As imaging and tumor characterization continues to evolve, the definition of tumor bulk may also need to be refined and incorporate volumetric metabolic information such as TLG [21]. In this study, the two variables selected by the LASSO method for our model represent a modern quantification of classically known risk factors: extranodal disease and tumor bulk. This model could be used to select patients at the highest risk for PD and death after CAR-T that may benefit from treatment intensification such as adjuvant systemic therapy or consolidative radiation therapy after CAR-T infusion. One-month post-CAR-T imaging may also aid in this decision-making [14, 22].

Dean and colleagues demonstrated the prognostic significance of MTV on PET/CT prior to axi-cel infusion, with greater MTV associated with increased risk of PD and death [9]. In contrast, our analysis did not find any association between single time point MTV and risk of PD or death- only the dynamic metabolic change in PET/CT characteristics from pre-leuk to pre-LD was prognostic. Similar to our study, Wang and colleagues did not demonstrate an association between single time point MTV and OS, though they had a small study size of 19 patients. Similar to our findings, they correlated higher pre-CAR-T MTV with higher grade toxicity after CAR-T infusion [13]. Figura and colleagues identified pre-infusion clinical and radiographic factors associated with the risk of relapse after CAR-T, including SUVMax≥10 at the pre-LD PET, but did not assess MTV or TLG [23].

Pre-LD PET/CT characteristics were stronger predictors of duration of CRS and grade 3 + ICANS than pre-leuk PET/CT characteristics in this analysis, potentially because the pre-LD PET/CT is closer to the time of infusion and better represents the disease state at infusion. Patients with greater pre-LD total MTV had higher risk of grade 3 + ICANS, use of tocilizumab, and use of corticosteroids. These patients should be carefully monitored following infusion, and potentially considered for prophylaxis.

While this study provides clinically useful information to help inform prognosis and potentially identify patients who may benefit from treatment intensification, it has several limitations. First, this study includes only 69 patients treated at a single center, and external validation is needed and planned. Second, while the value of metabolic characteristics such as MTV and TLG are increasingly appreciated, they require specialized software and training, is often time intensive to collect this data and is thus not commonly calculated in clinical practice. Automation and standardization are needed in this field to generalize utility. Third, given the novelty of CAR-T therapy, follow-up remains relatively short in this study. Finally, NHL patients receiving CAR-T are a heterogeneous group, and prognostic factors may vary between NHL patients with different histologies, molecular characteristics, and prior treatment histories. Future studies are needed to improve upon this work with hopes to better stratify patients and guide management.

### Supplementary information

Supplemental Table 1

## Data Availability

The datasets generated during and/or analyzed during the current study are available from the corresponding author upon reasonable request.

## References

[1] Crump M, Neelapu SS, Farooq U, Van Den Neste E, Kuruvilla J, Westin J (2017). Outcomes in refractory diffuse large B-cell lymphoma: results from the international SCHOLAR-1 study. Blood.

[2] Locke FL, Ghobadi A, Jacobson CA, Miklos DB, Lekakis LJ, Oluwole OO (2019). Long-term safety and activity of axicabtagene ciloleucel in refractory large B-cell lymphoma (ZUMA-1): a single-arm, multicentre, phase 1-2 trial. Lancet Oncol.

[3] Crump M, Kuruvilla J, Couban S, MacDonald DA, Kukreti V, Kouroukis CT (2014). Randomized comparison of gemcitabine, dexamethasone, and cisplatin versus dexamethasone, cytarabine, and cisplatin chemotherapy before autologous stem-cell transplantation for relapsed and refractory aggressive lymphomas: NCIC-CTG LY.12. J Clin Oncol.

[4] Neelapu SS, Locke FL, Bartlett NL, Lekakis LJ, Miklos DB, Jacobson CA (2017). Axicabtagene Ciloleucel CAR T-cell therapy in refractory large B-cell lymphoma. N. Engl J Med.

[5] Schuster SJ, Bishop MR, Tam CS, Waller EK, Borchmann P, McGuirk JP (2019). Tisagenlecleucel in adult relapsed or refractory diffuse large B-cell lymphoma. N. Engl J Med.

[6] NCCN Guidelines Versoin 4.2021: B-Cell Lymphomas: National Comprehensive Cancer Network; 2021 [Available from: https://www.nccn.org/professionals/physician_gls/pdf/b-cell.pdf.

[7] Shah NN, Nagle SJ, Torigian DA, Farwell MD, Hwang WT, Frey N (2018). Early positron emission tomography/computed tomography as a predictor of response after CTL019 chimeric antigen receptor -T-cell therapy in B-cell non-Hodgkin lymphomas. Cytotherapy.

[8] Im HJ, Bradshaw T, Solaiyappan M, Cho SY (2018). Current methods to define metabolic tumor volume in positron emission tomography: which one is better?. Nucl Med Mol Imaging.

[9] Dean EA, Mhaskar RS, Lu H, Mousa MS, Krivenko GS, Lazaryan A (2020). High metabolic tumor volume is associated with decreased efficacy of axicabtagene ciloleucel in large B-cell lymphoma. Blood Adv.

[10] Malek E, Sendilnathan A, Yellu M, Petersen A, Fernandez-Ulloa M, Driscoll JJ (2015). Metabolic tumor volume on interim PET is a better predictor of outcome in diffuse large B-cell lymphoma than semiquantitative methods. Blood Cancer J.

[11] Song MK, Yang DH, Lee GW, Lim SN, Shin S, Pak KJ (2016). High total metabolic tumor volume in PET/CT predicts worse prognosis in diffuse large B cell lymphoma patients with bone marrow involvement in rituximab era. Leuk Res.

[12] Guo B, Tan X, Ke Q, Cen H (2019). Prognostic value of baseline metabolic tumor volume and total lesion glycolysis in patients with lymphoma: a meta-analysis. PLoS ONE.

[13] Wang J, Hu Y, Yang S, Wei G, Zhao X, Wu W (2019). Role of fluorodeoxyglucose positron emission tomography/computed tomography in predicting the adverse effects of chimeric antigen receptor T cell therapy in patients with non-Hodgkin lymphoma. Biol Blood Marrow Transpl.

[14] Breen WG, Hathcock MA, Young JR, Kowalchuk RO, Bansal R, Khurana A (2022). Metabolic characteristics and prognostic differentiation of aggressive lymphoma using one-month post-CAR-T FDG PET/CT. J Hematol Oncol.

[15] Cheson BD, Fisher RI, Barrington SF, Cavalli F, Schwartz LH, Zucca E (2014). Recommendations for initial evaluation, staging, and response assessment of Hodgkin and non-Hodgkin lymphoma: the Lugano classification. J Clin Oncol.

[16] Abramson JS, Gordon LI, Palomba ML, Lunning MA, Arnason JE, Forero-Torres A (2018). Updated safety and long term clinical outcomes in TRANSCEND NHL 001, pivotal trial of lisocabtagene maraleucel (JCAR017) in R/R aggressive NHL. J Clin Oncol.

[17] Locke FL, Miklos DB, Jacobson CA, Perales MA, Kersten MJ, Oluwole OO (2022). Axicabtagene ciloleucel as second-line therapy for large B-cell lymphoma. N. Engl J Med.

[18] Ziepert M, Hasenclever D, Kuhnt E, Glass B, Schmitz N, Pfreundschuh M (2010). Standard International prognostic index remains a valid predictor of outcome for patients with aggressive CD20+ B-cell lymphoma in the rituximab era. J Clin Oncol.

[19] Federico M, Bellei M, Marcheselli L, Luminari S, Lopez-Guillermo A, Vitolo U (2009). Follicular lymphoma international prognostic index 2: a new prognostic index for follicular lymphoma developed by the international follicular lymphoma prognostic factor project. J Clin Oncol.

[20] Held G, Murawski N, Ziepert M, Fleckenstein J, Poschel V, Zwick C (2014). Role of radiotherapy to bulky disease in elderly patients with aggressive B-cell lymphoma. J Clin Oncol.

[21] Barrington SF, Mikhaeel NG, Kostakoglu L, Meignan M, Hutchings M, Mueller SP (2014). Role of imaging in the staging and response assessment of lymphoma: consensus of the International Conference on Malignant Lymphomas Imaging Working Group. J Clin Oncol.

[22] Al Zaki A, Feng L, Watson G, Ahmed S, Mistry H, Nastoupil LJ, et al. Day 30 SUVmax predicts progression in lymphoma patients achieving PR/SD after CAR T-cell therapy. Blood Adv. 2022. 10.1182/bloodadvances.2021006715 PMC9092420 35015825

[23] Figura NB, Robinson TJ, Sim AJ, Wang X, Cao B, Chavez JC, et al. Patterns and predictors of failure in recurrent or refractory large B-cell lymphomas following chimeric antigen receptor (CAR) T-cell therapy. Int J Radiat Oncol Biol Phys. 2021. 10.1016/j.ijrobp.2021.06.038 PMC9791939 34242714

